# Defining a patient-centered approach to cancer survivorship care: development of the patient centered survivorship care index (PC-SCI)

**DOI:** 10.1186/s12913-021-07356-6

**Published:** 2021-12-18

**Authors:** K. Holly Mead, Yan Wang, Sean Cleary, Hannah Arem, Mandi L. Pratt-Chapman

**Affiliations:** 1 Milken Institute School of Public Health, George Washington University, 950 New Hampshire Ave, Washington, DC 20052 USA; 2 Healthcare Delivery Research, MedStar Health Research Institute, Washington, DC 20008 USA; 3 Department of Oncology, Georgetown Medical School, Washington, DC 20007 USA; 4 George Washington University Cancer Center, George Washington University, 2600 Virginia Ave, NW, #300, Washington, DC 20037 USA

**Keywords:** Patient-Centered, Survivorship Care, Index, Measures, Confirmatory Factor Analysis

## Abstract

**Purpose:**

This study presents the validation of an index that defines and measures a patient-centered approach to quality survivorship care.

**Methods:**

We conducted a national survey of 1,278 survivors of breast, prostate, and colorectal cancers to identify their priorities for cancer survivorship care. We identified 42 items that were “very important or absolutely essential” to study participants. We then conducted exploratory and confirmatory factor analyses (EFA/CFA) to develop and validate the Patient-Centered Survivorship Care Index (PC-SCI).

**Results:**

A seven-factor structure was identified based on EFA on a randomly split half sample and then validated by CFA based on the other half sample. The seven factors include: (1) information and support in survivorship (7 items), (2) having a medical home (10 items) (3) patient engagement in care (3 items), (4) care coordination (5 items), (5) insurance navigation (3 items), (6) care transitions from oncologist to primary care (3 items), and (7) prevention and wellness services (5 items). All factors have excellent composite reliabilities (Cronbach’s alpha 0.84-0.94, Coefficient of Omega: 0.81-0.94).

**Conclusions:**

Providing quality post-treatment care is critical for the long-term health and well-being of survivors. The PC-SCI defines a patient-centered approach to survivorship care to complement clinical practice guidelines. The PC-SCI has acceptable composite reliability, providing the field with a valid instrument of patient-centered survivorship care. The PC-SCI provides cancer centers with a means to guide, measure and monitor the development of their survivorship care to align with patient priorities of care.

**Trial registration:**

ClinicalTrials.gov ID: NCT02362750, 13 February 2015

**Supplementary Information:**

The online version contains supplementary material available at 10.1186/s12913-021-07356-6.

## Introduction

Americans diagnosed with cancer today have a nearly seven in 10 chance of surviving 5 years, leaving a substantial population with unique health care needs. In 2019, there were nearly 17 million cancer survivors (defined from the moment of diagnosis) and this number is projected to grow to 21.7 million by 2030 [1, 2]. Cancer survivors^1^ suffer myriad physical and psycho-social consequences of cancer and its treatment, including pain, fatigue, anxiety, and depression, as well as practical concerns such as health insurance challenges and financial hardships. While some of these issues are short-lived, late-emerging and long-term effects, such as neurological sequalae, fatigue, cardiomyopathy, fertility issues, and long-term emotional distress, affect many survivors for years [3–6].

The unique challenges of cancer survivorship have created the need for a distinct, post-treatment phase of cancer care that shifts the focus from treating the disease to managing the chronic effects of cancer and focusing on the quality of life of survivors over the long term [3, 7, 8]. In response to this need, the National Policy Forum of the National Academies of Sciences, Engineering and Medicine (NASEM) issued a call to action in 2006 and the Commission on Cancer began requiring focused attention on the post-treatment care of cancer survivors [9]. Subsequently, a variety of care models have arisen, including nurse-led models [10, 11], primary care models [12], models in which oncology and primary care share different aspects of post-treatment care [13], transitional care models [14], and—most recently--virtual care models [15]. As survivorship care programs work to address the needs of this growing patient population, cancer researchers and providers are in need of guidelines and strategies to clearly define, measure and implement high quality, patient-centered survivorship care across all models of care [6, 9].

Cancer survivorship care should encompass all follow-up care, referrals and resources needed by survivors to manage their long-term health, including monitoring and surveillance for new and returning cancers, preventive and risk-management services, care for ongoing and late-emerging physical symptoms, management of co-morbidities, support for emotional issues, and help with financial and practical concerns [3, 16]. Moreover, because of the heterogenous impact of cancer on patients’ health and well-being, survivorship care should also offer a patient-centered approach that prioritizes patients’ individual preferences and needs to optimize their understanding of and engagement in their care. Studies suggest that patient-centered care can enhance quality of care and improve clinical outcomes [17–22]. When patients’ priorities are met, they experience higher self-efficacy and better self-management of their care, greater adherence to health promotion strategies, decreased suffering and symptom burden, and improved quality of life [17, 23]. Much of the effort to improve quality cancer care for survivors in the U.S, has focused on the implementation of important clinical metrics, however, patient-centered strategies that directly address the practices and interactions that ensure patients’ priorities for care are being met may be overlooked.

Patient-centered care in cancer survivorship is a concept that has not been fully developed or translated into clinical practice. A number of studies have examined patients’ preferences and found a need for greater awareness and information about life after cancer treatment, tools to engage patients in their care, improved communication between providers and patients, increased access to risk-management and health promotion services, and better care coordination, particularly around transitions in care [23–28]. Some studies outside the U.S. have developed patient-centered measures for cancer care, setting precedent for the importance of integrating patient-centered care into clinical guidance. Few studies looking at the U.S. cancer care system, however, have examined how to holistically address patient-centered survivorship needs in practice, and, to our knowledge, there are no extant tools that measure and evaluate the multiple dimensions of patient-centeredness in survivorship care from patients’ perspectives, although a recently published framework identifies important elements of care as defined by survivorship experts [24, 29, 30].

The purpose of this study is to develop and validate a survivorship care index to more clearly define and operationalize a patient-centered approach to survivorship care. The Patient-Centered Survivorship Care Index (PC-SCI) provides a systematic approach to identifying and implementing practices that advance survivors’ priorities for post-treatment cancer care. Moreover, the PC-SCI offers cancer survivorship care providers a framework to organize high quality, patient-centered survivorship care and a tool to assess how well survivorship care meets patients’ goals and values. We hypothesized that there were several different latent constructs underlying the cancer patients’ survivorship care priorities. This study was registered on ClinicalTrials.gov, ID: NCT02362750, on13 February 2015.

## Methods

### Study approach

We developed the PC-SCI as part of a larger comparative effectiveness research (CER) project, Evaluating Cancer Survivorship Care Models, which examined the impact of different models of survivorship care on patient outcomes.^2^ The PC-SCI was the primary measure of patient-centered care in the CER study and was used to evaluate the patient-centered approach of 32 survivorship care programs across the United States and examine their effectiveness in addressing survivors’ sense of self-efficacy, health care utilization, and physical and emotional quality of life [31].

In the formative phase, we conducted a qualitative study comprising 22 focus groups with 170 breast, prostate and colorectal cancer survivors to identify patients’ priorities for survivorship care. The PC-SCI was developed based on the results of this work, which identified care practices that participants characterized as patient-centered priorities for survivorship care. Information on the methodology and results of the qualitative formative work have been reported elsewhere [24]. In this phase of the research, we conducted a national survey of breast, prostate and colorectal cancer patients to develop and validate the PC-SCI using exploratory and confirmatory factor analysis (EFA/CFA). The George Washington University Institutional Review Board approved the study (#101308).

### Survey development

Following the qualitative analysis, we conducted a national survey of breast, prostate and colorectal cancer survivors to further examine their perspectives on patient-centered survivorship care and identify specific practice items that survivors consider to be most important in survivorship care. The objectives of the survey were to develop and validate a multiple-factor index that represents survivors’ priorities for care. The survey was developed around the practice priorities identified in the qualitative analysis and reflected survivors’ preferences for survivorship care. We limited the survey sample to survivors of the three most common cancers to facilitate sampling and ensure we had a large enough sample to conduct the analysis. We also were interested in examining common experiences among survivors of different types of cancers to better understand how survivorship care can be better organized to address all survivors’ needs, which we did in the formative work.

Most items in the survey were adapted from publicly available, validated instruments with related constructs around components of patient-centeredness and quality [32–40]. We did not find an instrument specific to survivors desire for a patient-centered medical home (PCMH) and, instead drew from the literature and guidance, an organizational PCMH assessment tool and guidance from a community advisory board (CAB) comprised of cancer survivor and cancer survivorship care provider stakeholders to develop new items for this concept. We conducted 15 cognitive interviews to test item readability and comprehension, appropriateness of response categories, correct frame of reference, and understanding of specific terminology. The final survey consisted of a total of 68 items representing specific services and practices that operationalize patient-centered care priorities from the formative work. Using a 5-point Likert scale, survey questions asked survivors to report on the patient-centeredness of care by rating “how important it is” to receive elements of care related to psychosocial support, information and resources, self-management, clinician support, clinician-patient communication, care coordination, holistic care, practical life support and having a medical home. The range of the 5-point Likert scale was as follows: 1=not important, 2=somewhat important, 3=important, 4=very important and 5=absolutely essential.

### Sample design and survey administration

We contracted with GfK Knowledge Networks to field an internet-based survey from August-September of 2014. The study population was a blended sample consisting of GfK’s KnowledgePanel® (KP), a probability-based web panel, and a non-probability opt-in sample, to oversample participants who had been diagnosed with cancer and to achieve a proportionate distribution of breast, prostate and colorectal cancer survivors. Eligible respondents were non-institutionalized adults ages 18 and older in the U.S. who had been diagnosed with breast, prostate or colorectal cancer and had completed active treatment at any point in time. GfK used a calibrated weighting system^3^ to ensure the blended sample was representative of our target population. We targeted a sample size of 1,270 to ensure precision in our statistical estimates. The estimated sample size needed for the survey was based on estimates of the proportion of the sample endorsing each element of quality and the magnitude of the differences between the ranked elements. A small effect size (*f* = .10) was estimated to be a 10-15% difference. The target sample size for the web-based survey was 1,269 with alpha = 0.05 and 90% power. The survey was disseminated to 97,329 panelists in the blended panel, and data were collected until the desired sample was achieved. Given GfK’s sampling strategy of using a pre-enrolled probability-based panel for surveys, the firm reports completion rates rather than response rates; completion rates reflect the percent of panelists, who completed the preliminary screening survey and main survey. Table 1 presents the number of respondents in both the probability-based web panel and the non-probability opt-in sample. A total of 1,278 participants were eligible for and completed the survey.

**Table 1:** National survivorship care survey participant characteristics

Characteristic	***n*** = 1278
*Age, M*	
Mean	64
*Gender, N (%)*	
female	719 (56.3)
*Cancer site, N (%)*	
Breast	679 (53.1)
Prostate	451 (35.3)
Colorectal	148 (11.6)
*Stage at diagnosis, N (%)*	
Stage 0	157 (12.3)
Stage 1	385 (30.1)
Stage 2	227 (17.8)
Stage 3	227 (9.8)
Stage 4	36 (2.8)
Don’t Know	345 (31)
*Time since cancer diagnosis, N (%)*	
<1 year	38 (3.0)
1-10 years	800 (62.7)
11-20 years	341 (26.7)
21-30 years	79 (6.2)
31-40 years	16 (1.3)
41-50 years	2 (0.0)
*Treatment, N (%)*	
Surgery	1033 (80.8)
Chemotherapy	433 (33.9)
Radiation	637 (49.8)
Hormonal therapy	398 (31.1)
Other	81 (6.3)
*Race, N (%)*	
White	1099 (86)
Black	63 (4.9)
Other	50 (3.9)
*Hispanic ethnicity, N (%)*	
yes	66 (6.2)
*Insurance status N (%)*	
yes	1259 (98.5)
*Insurance type*	
Private	642 (50.2)
Medicare	783 (61.3)
Medicare supplement	389 (30.4)
Medicaid	85 (6.7)
Other	54 (4.2)

*Note*: Percentages are calculated based on valid counts and exclude missing data

To determine survivors’ most important priorities for survivorship care, survey participants were asked to reflect on the 68 survivorship care items in the survey and score each items’ importance. Items were classified as critical to patients’ survivorship care when: (1) the majority (≥50%) of respondents reported the item as either “3=important,” “4=very important,” or “5=absolutely essential” and (2) at least ≥30 percent indicated the item as “4=very important” or “5=absolutely essential.”

### Statistical analyses

We conducted exploratory factor analyses (EFA) to identify factors and items for the PC-SCI and then confirmatory factor analysis (CFA) to validate the factor structure. The sample of survey respondents was randomly split into testing (*n* = 639) and validation (*n* = 639) groups. EFA was run first with no predetermined factor structure and no restrictions to explore the factor structure within half of the sample. The suitability of factor analysis was verified by calculating the Kaiser-Meyer-Olkin (KMO) index of sampling adequacy (> .80) [41] and Bartlett’s test of sphericity (with *p* < .05) [42]. The Robust Maximum Likelihood (RML) estimation method, with an oblique rotation (Geomin) [43], was used because we anticipated moderate correlations between factors and to account for possible non-normal distributions of the items. For the EFA, the number of factors was determined based on several criteria: (1) the Kaiser’s criteria of retaining all factors with eigenvalues>=1 [44, 45] (2) Cattell’s scree plot based on eigenvalues and retaining the factors before the break point in the line [46], (3) Hom’s parallel analysis that retains the number of factors at the crossing point of the screen plot and eigenvalues estimated from a set of random variables [47, 48] and (4) theoretical meaningfulness. Then confirmatory factor analysis (CFA) was conducted based on the results of EFA to validate the factor structure. For the model fit of the CFA, standard fit was examined to identify the final model statistics, including RMSEA, CFI, TLI, SRMR. X^2^ test was sensitive to sample size and is biased with a large sample size. RMSEA<0.08, CFI>0.9 and SRMR<0.08 were deemed acceptable fit [49]. Missing items were treated with full information maximum likelihood (FIML) in the EFA and CFA models. The internal consistency for each factor was assessed with Cronbach’s alpha and coefficient omega, separately, with high values indicating high reliabilities [50]. All the analyses accounted for the sampling weight in the survey. The research team conducted EFA/CFA using Mplus 8.4 statistical software and KMO and Bartlett’s tests were conducted with STATA 16 statistical software.

## Results

### Sample characteristics

The average age of survey participants was 64 years, and 56 percent were female (Table 1). Just over half (53 percent) had been diagnosed with breast cancer, 35 percent with prostate cancer, and 12 percent with colorectal cancer. The majority of participants self-reported as White (86 percent), five percent self-reported as African American, and six percent as Hispanic. Over half (63 percent) of the sample had been diagnosed within one to 10 years and 27 percent had been diagnosed with cancer within 11 to 20 years of the survey. Nearly 99 percent had some form of insurance.

### Content validity

Forty-two survey items were determined to be patient-centered priorities for survivorship care based on the threshold of ≥30 percent of participants scoring them as “very important or absolutely essential” (see Table 2). The 42 items were reviewed by the CAB and the research team to assess content validity. The research team eliminated four items due to redundancy in content, leaving 38 items for the EFA (See Table 2). The absolute values for skewness of each item ranged within 2, and the absolute values for kurtosis ranged within 3.

**Table 2:** National survivorship care survey items by priority practice

Survey items measuring “During survivorship care, how important is it that you”	Percent rating very important ^a^
Participate in decision-making about your cancer-related follow-up care	81.3
Know all clinicians involved in care know your medication	79.6
Feel in control of health and can manage follow-up care to improve health	76.7
Receive referrals for cancer-related follow-up care	76.0
Know all clinicians have your medical files on cancer care	75.0
Know all clinicians share information with each other to stay up-to-date about your follow-up care	74.2
Regularly receive complete physical with medical history	71.9
*Receive referrals to non-cancer specialty and follow-up services*	70.0
Are informed about health problems and how to take care of them	66.9
Receive written survivorship care plan with recommendations for follow up care	63.5
Receive written treatment summary^c^	62.3
Know all clinicians can access medical records online or through EHR	60.4
Receive courtesy and respect	60.0
Have regular access to risk reduction programs (e.g. weight loss, smoking cessation^c^)	59.3
Discuss screening needs and recommendations for follow up care	59.0
Have regular clinician/place to get all follow-up needs met	58.4
Receive support to manage relationships with partners and family	58.2
Have concerns related to cancer after treatment listened to	58.0
Have enough time to ask questions/voice concerns during visits	57.4
Have help understanding insurance coverage options for medical services	56.4
Have help with insurance problems, e.g. rejected claims	56.4
Receive explanation in a way that is easy to understand about follow-up care	55.3
Discuss late/long-term side effects of cancer and treatment	54.1
Receive support to manage what life is like after treatment ends	53.1
Have help understanding insurance coverage options for Rx and OTC drugs	53.1
Receive explanation for medical tests related to follow-up care	53.0
*Remain under cancer clinician until ready to transfer care is discussed*	52.4
Receive referrals to mental health care providers	51.8
Discuss emotional concerns with regular doctor during follow-up care	51.6
*Discuss preference for treatment clinician to oversee post-treatment survivorship care*	51.0
Have team of clinicians who all work together to address follow-up health care	51.0
Receive help problem-solving new health care issues	50.4
Access your own medical records and recommendations online or through EHR	50.3
*Discuss preference for transferring care to PCP*	49.3
Have regular access to exercise and physical activity services	49.0
Know cancer clinician stays informed of your health after care is transferred	48.0
Have a point of contact to answer questions/concerns about follow-up care	47.0
*Decide with clinician when/how to transition from or share care between oncologist and PCP*	46.0
Receive instructions on when and how to transition care from oncologist back to PCP	45.0
Receive help with follow through on recommendations for follow-up	42.6
*Receive information and guidance on who to call when experiencing health problems*	42.5
Have regular access to nutrition and dietary services	40.1

Note: *Italicized* items were not included in the EFA due to redundant content or low factor loading

### Construct validity

The data showed good sampling adequacy (KMO>0.9) and Bartletts’s test of sphericity was significant (x2=16973, df=703, *p*<0.001). In the 38-item EFA, we analyzed models with increasing factors from 1-factor to 12-factors using Geomin rotation with robust maximum likelihood. Different models were preferred based on our evaluation criteria; the eigenvalues suggested a 6-factor model, while the scree plot suggested a 7-factor model and the parallel analysis suggested a 3-factor model (3 factors having eigenvalues greater than the random median eigenvalues). The study team compared the three models and determined the 6-factor and 3-factor models had multiple cross-loadings, and that neither had clear factor interpretations. We, therefore, chose the 7-factor model because it had only one item with cross-loadings and the factors made theoretical sense.

A factor loading ≥0.4 was used for a cutoff. Two items (“Receive instructions on when and how to transition care from oncologist back to PCP”, and “Clinician explains things about cancer follow up care in a way that is easy to understand”) had weaker factor loadings (0.38 and 0.37, separately), but because they were close to the cutoff and conceptually important, the study team chose to retain them in the model. One item (“Patient has regular access to exercise and physical activity services”) had cross-loadings on two factors (“Patient engagement” and “Prevention and wellness services”), and the study team categorized the item based on the higher loading. Two items (“Clinician can provide referrals to non-cancer specialty and follow-up services” and “Clinician provides information and guidance on who to call when experiencing health problems”) had weak factor loadings (<.35) and were therefore dropped from the model. In total, 36 items were included in the CFA analyses.

CFA was conducted to validate the model and each item was expected to load onto a single factor. Initially the CFA model did not meet the satisfactory fit of the data (X^2^=1637.17, df=573, *p*<0.001, RMSEA=0.054, CFI: 0.88, TLI:0.87, SRMR: 0.06). Based on the modification indices, we revised the CFA model and included 4 residual covariance between items (see Table 3). Three covariances can be explained by either similar wording (e.g. “Clinician discusses screening needs and recommendations for follow up” and “Clinician discusses late/long-term effects of cancer and treatment”; “Clinician provides patient with written treatment summary” and “Clinician provides patient with written survivorship care plan with recommendations for follow up care”; “Patient has regular access to exercise and physical activity services” and “Patient has regular access to nutrition and dietary services”) and the fourth can be explained due to correlated content (e.g. “Patient has enough time to ask questions/voice concerns during visits” and “Clinician listens carefully to concerns related to patient health that may be related to cancer after treatment”). The final CFA model shows satisfactory model fit (X^2^=1356.52, df=569, *p*<0.001, RMSEA=0.047, CFI: 0.91, TLI: 0.90, SRMR: 0.06). Except for one item with a factor loading of 0.47 and another item with a factor loading of 0.56, all other factor loadings were greater than 0.6. The Cronbach’s alpha ranges within 0.84-0.94. The coefficient omega ranges within 0.81-0.94, indicating high reliability for each factor. All 7 factors are highly correlated with the correlation coefficient ranging within 0.50-0.90 (ps<0.001). When the same CFA model was applied to the total sample, the model fit was greatly improved (X^2^=1712.461, df=569, *p*<0.001, RMSEA=0.040, CFI: 0.93, TLI: 0.92, SRMR: 0.05).

**Table 3:** Final confirmatory factor analysis model

Factors and items			
**Factor 1. Information and support in survivorship**	Factor-based score: Mean (SD): 3.38 (1.13), Cronbach’s α= 0.93, McDonald's omega =0.93, AVE=0.66		
	Standardized factor loading	Standardized residual variance (1-R2)	Explained variance (R2)
**Clinician discusses screening needs and recommendations for follow up care**^a^	0.81 (0.02)	.338	.662
**Clinician discusses late/long-term side effects of cancer and treatment**^a^	0.83 (0.03)	.318	.682
Clinician helps patient follow through on recommendations for follow-up	0.81 (0.03)	.340	.660
**Clinician provides patient with written treatment summary**^b^	0.76 (0.03)	.422	.578
**Clinician provides patient with written survivorship care plan with recommendations for FU care**^b^	0.81 (0.02)	.340	.660
Clinician and patient decide together when and how to transition from oncologist to PCP	0.82 (0.03)	.327	.673
Clinician explains things about cancer follow up care in a way easy to understand	0.84 (0.02)	.296	.704
**Factor 2. Medical home**	Factor-based score: Mean (SD): 3.53 (0.96), Cronbach’s α=0.94, McDonald’s omega=0.94, AVE=0.62		
	Standardized factor loading	Standardized residual variance (1-R^2^)	Explained variance (R^2^)
Clinician explains reason for medical tests related to follow-up care	0.80 (0.04)	.359	.641
Clinician addresses patient emotional concerns when discussing follow-up care	0.72 (0.03)	.481	.519
**Patient has enough time to ask questions/voice concerns during visits**^c^	0.82 (0.02)	.327	.673
**Clinician listens carefully to concerns related to patient health that may be related to cancer after treatment**^c^	0.84 (0.02)	.296	.704
Clinician shows respect for what patient has to say about follow up care	0.83 (0.02)	.304	.626
Clinician helps figure out reasons for new health care problems and whether they are related to cancer	0.80 (0.04)	.355	.645
Patient has regular clinician/place to get healthcare needs met including follow-up care	0.84 (0.02)	.297	.703
Patient has a point of contact to answer questions/concerns about follow-up care	0.84 (0.02)	.291	.709
All clinicians can access medical records online or through EHR	0.67 (0.04)	.547	.453
Patient has team of clinicians who all work together to address follow-up health care	0.68 (0.04)	.543	.457
**Factor 3. Patient engagement**	Factor-based score: Mean (SD): 4.14 (0.82) Cronbach’s α=0.86, McDonald’s omega =0.86 AVE=0.67		
	Standardized factor loading	Standardized residual variance (1-R2)	Explained variance (R2)
Patient is included in decision-making about your cancer-related follow-up care	0.72 (0.04)	.479	.521
Patient is informed about what to do every day to take care of health and healthcare needs	0.88 (0.02)	.221	.779
Patient feels in control of health and can manage health care needs	0.84 (0.03)	.303	.697
**Factor 4. Care coordination**	Factor-based score Mean (SD): 4.17 (0.86) Cronbach’s α=0.90, McDonald’s omega =0.91 AVE=0.66		
	Standardized factor loading	Standardized residual variance (1-R2)	Explained variance (R2)
All clinicians involved in care know patient’s medication	0.83 (0.02)	.314	.686
All clinicians have medical files on cancer care	0.89 (0.02)	.205	.795
All clinicians share information with each other about follow-up care	0.85 (0.03)	.284	.716
Clinicians can give patient the name of a doctor to make an appointment with if they need more follow-up care	0.88 (0.02)	.221	.779
Patient receives complete physical exam with medical history	0.56 (0.05)	.685	.315
**Factor 5. Help navigating insurance**	Factor-based score Mean (SD): 3.59 (1.11) Cronbach’s α=0.89, McDonald's omega =0.89 AVE=0.73		
	Standardized factor loading	Standardized residual variance (1-R2)	Explained variance (R2)
Patient has help understanding insurance coverage options for medical services	0.86 (0.02)	.266	.734
Patient has help understanding insurance coverage options for Rx and OTC drugs	0.92 (0.02)	.161	.839
Patient has help with insurance problems, e.g. rejected claims	0.79 (0.04)	.373	.627
**Factor 6. Care transitions**	Factor-based score Mean (SD): 3.49 (1.12) Cronbach’s α=0.86, McDonald's omega =0.88 AVE=0.71		
	Standardized factor loading	Standardized residual variance (1-R2)	Explained variance (R2)
Receive instructions on when and how to transition care from oncologist back to PCP	0.82 (0.02)	.324	.676
Patient remains under care of cancer doctor until ready to move care back to primary care doctor	0.87 (0.03)	.237	.763
Cancer doctor stays informed of patient health after patient transfers care to primary care doctor	0.83 (0.03)	.311	.689
**Factor 7. Prevention and wellness services**	Factor-based score Mean (SD): 3.06 (1.00) Cronbach’s α=0.84, McDonald's omega =0.81 AVE=0.45		
	Standardized factor loading	Standardized residual variance (1-R2)	Explained variance (R2)
**Patient has regular access to exercise and physical activity services**^d^	0.47 (0.05)	.781	.219
**Patient has regular access to nutrition and dietary services**^d^	0.63 (0.05)	.606	.394
Patient has regular access to risk reduction programs (e.g. weight loss, smoking cessation)	0.72 (0.04)	.482	.518
Clinicians can provide referrals to patient for mental health effects related to cancer treatment	0.76 (0.03)	.423	.577
Patient has support to manage roles and relationships with partner, family, and others	0.75 (0.03)	.436	.564

*Note*: 1. All standardized factor loadings are significantly different from zero (ps<0.001). 2. There are four pairs of residual error correlations with the corresponding items in bold: the correlation coefficient (r) between the residual errors of two items marked with ^a^ is 0.375 (*p*<0.001), r for the two items marked with ^b^ is 0.491 (*p*<0.001), r for the two items marked with ^c^ is 0.389 (*p*<0.001) and r for the items marked with ^d^ is 0.615 (*p*<0.001). All the factors are positively correlated with the correlation coefficient ranging within 0.498-0.828 (all ps<0.001). The model fitness criteria: Chi-square: 1356.52(569), *p*<0.001, RMSEA: 0.047, CFI: 0.911, TLI: 0.902, SRMR:0.060. Factor-based score was created based on the mean of the scores of the items within each factor, accounting for sampling weight

In the final CFA, the seven factors are: (1) information and support in survivorship (7 items); (2) having a medical home (10 items); (3) patient engagement in care (3 items); (4) survivorship care coordination (5 items); (5) insurance navigation (3 items); (6) care transitions from oncologist to primary care (3 items); and (7) prevention and general wellness services (5 items). It is visually complex to present the 7 factors in a figure, so the factor structure is presented in Table 3 with standardized factor loadings and standard errors for each loading in the 2^nd^ column. The standardized residual errors are presented in the 3^rd^ column for each item. The R2 in column 4 indicates the standardized variance explained by the factors. Four pairs of items are bold to indicate residual correlations between the items ranging between 0.375 and 0.615. All the correlations between the latent factors are significant (ps<0.001), ranging within 0.498-0.828 (see Table 4).

**Table 4:** Correlation coefficients among the factor-based scores (factors 1-7)

	f1	f2	f3	f4	f5	f6	f7
**f1**	1.00						
**f2**	0.82	1.00					
**f3**	0.60	0.68	1.00				
**f4**	0.58	0.64	0.55	1.00			
**f5**	0.62	0.60	0.50	0.52	1.00		
**f6**	0.72	0.65	0.48	0.64	0.59	1.00	
**f7**	0.70	0.68	0.50	0.52	0.62	0.69	1.00

*Note*: f1-f7 indicate factor-based scores for factor 1 to factor 7 in Table 3

### Convergent validity

The convergent validity is measured by two methods: (1) reliability for each factor including Cronbach’s alpha and McDonald’s Omega, separately, and (2) the Average Variance Extracted (AVE) for each factor. The Cronbach’s alpha and McDonald’s Omega are greater than 0.7 for each of the latent factors. The AVE is calculated by getting the R2 value for each indicator in the CFA, adding them together and dividing by the total number of indicators [51]. The AVE for six factors are greater than 0.5 and AVE for one factor is close to 0.5, indicating convergent validity for the factors.

### Factor-based scores

The factor-based scores for each factor were created based on the average of the scores of the items within each factor in the CFA model with the mean (SD) accounting for sampling weight shown in Table 3. The average score was estimated instead of the summary score as it has the advantage of being on the same scale for each factor and does not vary by the number of items. Only factors 1 and 2 show higher correlation (.82), likely due to the importance of communication in both factors (i.e., Information and support in survivorship and Medical Home).

## Discussion

More can be done to ensure a comprehensive patient-centered approach to care that delivers the services and practices that help cancer survivors achieve better clinical outcomes and quality of life. In this study, we developed and validated a patient-centered survivorship care index that can be used to guide the development of survivorship programs and measure the patient-centeredness of care. The PC-SCI comprises seven multi-item factors that reflect cancer survivors’ priorities for survivorship care: Information and support in survivorship, medical home, patient engagement, care coordination, help navigating insurance, care transitions, and prevention and wellness services. These factors represent patient-centered resources and services that have been reported by patients to be priorities for survivorship care. Furthermore, the seven factors validated in the PC-SCI are well established in the literature as concepts critical to the provision of high-quality, patient-centered care, and should be leveraged to improve survivorship care and survivors’ long-term outcomes.

The PC-SCI highlights survivors’ desire for information and support to help with the long-term management of their post-treatment care. Studies show the importance of having well-informed patients who understand the trajectory of their health and wellness as a survivor and what to expect in the survivorship phase of their cancer care. When cancer treatment is completed, patients want a better understanding of “what happens next.” [24, 25] Uncertainty and lack of guidance may exacerbate patient challenges post-treatment [24, 25]. When care addresses information needs such as screening recommendations, care plans and treatment summaries, patients have clearer expectations, and they feel more prepared and grounded for the next stage in their health care [23, 24]. Provider support and help with follow-up also increase patients’ skill and confidence in making decisions and managing their care [26, 52].

The PC-SCI also highlights the patient-centered benefits of a medical home model for survivors, given its emphasis on the patient-provider relationship and patient-centered principles, such as communication and partnership between survivors and their clinicians, as well as the infrastructure support that enables continuity and coordination of care [28]. When providers utilize effective communication and shared decision-making and demonstrate respect, trust, and shared responsibilities with their patients, patients are more self-efficacious and have better physical and psychological health [20, 23]. Furthermore, strong communication and shared decision-making improve patients’ recovery, lessen symptoms, improve emotional health and support better overall quality of life [18, 53–55]. A medical home model for survivorship also typically takes a team-based approach with resources that facilitate access to continuous, follow-up care that can improve survivors’ overall health and well-being [56, 57].

Patient engagement is another key component of the PC-SCI. Patients who actively engage in decisions about their own care have been found to demonstrate higher self-efficacy, resulting in fewer barriers to care and improved clinical outcomes [21, 22, 26, 58]. Patient activation has also been shown to improve the uptake of preventive services and reduce risky behaviors [52]. The PC-SCI highlights survivors’ desire for better care coordination and transitions in survivorship care [25, 59]. Practices such as referral coordination, care management, and transition visits improve the patient experience, as well as the quality of care [20, 26, 60, 61]. With these practices in place, patient care is less fragmented and inconsistent, and may lower costs, reduce inappropriate care and improve health outcomes [56, 62]. Finally, the PC-SCI highlights the importance of a patient-centered, holistic approach to survivorship care that offers prevention and wellness services. Studies show patients want and need services such as mental health care referrals, dietary and exercise resources, and risk-reduction programs [9, 20, 25, 63, 64]. A patient-centered approach also recommends offering practical needs support, such as help navigating insurance [9, 24].

Future research should examine the use of the PC-SCI in clinical practice. A recommended approach would be to survey patients with item stem “During today’s visit” or “Following your visit today” or “On an ongoing basis, do you,” depending on the PC-SCI item. Responses should provide a range with a minimum of No/Yes/Don’t know/Don’t need or a range from Never to Always with a “Don’t know” and “Don’t need” option. This approach has been piloted among six state cancer coalitions and two tribal clinics using the Advancing Patient Centered Survivorship Care Toolkit, available online, which includes clinical support tools, workshop activities, assessment and evaluation tools based on the PC-SCI. See Supplemental Material for a formatted questionnaire. In addition, future studies might also use other statistical methods, e.g. Item Response Theory [65, 66], which can provide important information, e.g. item difficulty, discriminative ability, and might address other factor-related issues raised in this study.

As with all research, the current study has limitations that may affect the results. The survey consisted of self-reported data and is, therefore, subject to participant bias where respondents over- or under-report their experiences with certain items. In addition, while we aimed for a diverse cancer survivor sample, in line with other studies, our survey sample was mostly white, insured, had a history of breast cancer, with a mean age of 64. Therefore, more work needs to be done to validate the PC-SCI among more diverse groups, including diverse racial and ethnic groups, sexual and gender minorities, and those with a history of cancers other than breast cancer. In addition, priorities for patients’ survivorship care may differ by cancer types, treatment types or treatment phases, future studies might assess the constructs of survivorship care in the subpopulations of cancer survivors. Another limitation is that no related measures were included in this study for comparison. Future studies might include other existing survivorship care measures and assess the criterion validity of the survivorship care index by linking the indices to other existing measures.

Sampling and non-response bias, as well as survey error, are also challenges with all survey research. To address these concerns, the survey contractor GfK computed survey weights to offset known selection bias, including cancer type, and used a panel demographic post-stratification weight as an additional adjustment based on demographic distribution from the most recent Current Population Survey (CPS). While our statistical methods control for confounding factors, unaccounted influence could impact the results.

The survey was conducted in 2014 and much has evolved in cancer survivorship care since that time. However, issues related to how best to provide patient-centered survivorship care continue to challenge the field as noted by recent studies published by Hobden et al. and Tirodkar et al. [67, 68] These studies suggest gaps in patient-centered survivorship care still exist and more work is needed to support transformation of survivorship care programs to a truly patient-centered experience. Thus, the research team feels the PC-SCI is a relevant and useful contribution to the field as a tool that can help programs identify dimensions of patient-centeredness that can be improved upon.

Limitations related to the factor analysis could also affect the PC-SCI. While the research team aimed to develop a complete and accurate set of patient-centered practices in the survey by basing the work on theoretical models, conducting extensive formative work with survivors and using validated survey items [24], it is possible we missed important attributes, which could reduce the value of the index in measuring important aspects of patient-centered care.

## Conclusion

Patient-centered care is a principal component of high-quality care. In order for care to be most effective, it must address patients’ self-identified needs, respect their values, consider their preferences in decision-making and respond to their priorities for better health and wellness [69]. As the field of survivorship care matures and professional standards are developed in the U.S., it is important to offer guidance that ensures care is both clinically optimal and patient-centered [70]. The PC-SCI provides cancer center clinicians and researchers with a validated tool to guide, measure, and monitor efforts that align quality survivorship goals with patient priorities for care.

## Supplementary Information

**Additional file 1.**

## Abbreviations

CER: Comparative Effectiveness Research
CFA: Confirmatory Factor Analysis
CFI: Comparative Fit Index
EFA: Exploratory Factor Analysis
FIML: Full Information Maximum Likelihood
KMO: Kaiser-Meyer-Olkin index of sampling adequacy
RML: The Robust Maximum Likelihood
RMSEA: Root Mean Square Error of Approximation
SRMR: Standardized Root Mean Squared Residual
TLI: Tucker-Lewis Index

## Footnotes

1 A person is considered a cancer survivor from the time of diagnosis until the time of death [1]. However, because we are looking at the system of care provided to survivors post-treatment, we use the American Society of Clinical Oncology’s “functional definition,” which considers survivors to be those “individuals who have successfully completed curative treatment of those who have transitioned to maintenance of prophylactic therapy” [5].

2 Evaluating Cancer Survivorship Care Models was funded by the Patient-Centered Outcomes Research Institute (IH-12-11-5255).

3 The GfK calibration weighting allows the sample from the KP probability sample and the opt-in sample to be blended and better represent our study’s target population. It identifies how respondents from the two panels respond differently to specific questions and applies correcting weights to realign the opt-in panel respondents with the probability-based KP. This improved representation reflects basic geo-demographic distributions, as well as important attitudinal and behavioral measures. The methodology is widely used in published studies.
