# Supplementary material for: Defining a patient-centered approach to cancer survivorship care: development of the patient centered survivorship care index (PC-SCI)
Source: BMC Health Serv Res. 2021 Dec 18;21:1353. doi: 10.1186/s12913-021-07356-6 (PMC8684610; doi:10.1186/s12913-021-07356-6)
Supplement: Supplementary file 1 — Additional file 1. [file 12913_2021_7356_MOESM1_ESM.docx]

# **Cancer Survivor Survey**

How to use:

- Determine how long the survey will be open (e.g. 1 week, 2 weeks, etc.) in order to establish a baseline.
- Determine how survey will be administered (e.g. pen and paper, tablet, etc.).
- Ask survivors to complete the following survey after their visit.
- Aggregate findings to assess areas for quality improvement.

Sample Introductory Language:

*Thank you for taking the time to complete this survey about your visit today. [Organization name] is asking cancer survivors who have completed treatment to participate in this survey.*

*We would like to learn more about your experience with care after treatment and how we can improve your care. All your responses are anonymous and will not affect your care in any way. You can stop answering questions at any time.*

*This survey should take about 35 minutes to complete. Thank you again for your time.*

**Name: ___________________________________________________ Date: ____/____/________**

**1. During today’s visit:**

|  | No, never | No, but at another visit | Yes, briefly | Yes, in detail | Don’t know | | Don’t need this or N/A |
| --- | --- | --- | --- | --- | --- | --- | --- |
| Did you receive a complete physical exam with medical history? |  |  |  |  | |  |  |
| Did your clinician(s) discuss screening needs and recommendations for follow up care? |  |  |  |  |  | |  |
| Did your clinician(s) discuss late/long-term side effects of cancer and treatment? |  |  |  |  |  | |  |
| Did your clinician(s) address your emotional concerns when discussing follow-up care? |  |  |  |  |  | |  |
| Did you receive help understanding insurance coverage options for medical services? |  |  |  |  | |  |  |
| Did you receive help understanding insurance coverage options for prescription and over the counter drugs? |  |  |  |  | |  |  |
| Did you receive help dealing with insurance problems, e.g. rejected claims? |  |  |  |  | |  |  |
| Did your clinician(s) provide you with a written treatment summary? |  |  |  |  |  | |  |
| Did your clinician(s) provide you with a written survivorship care plan with recommendations for follow up care? |  |  |  |  |  | |  |

**2. During today’s visit:**

|  | Not at all | A little | Mostly | Completely | Don’t know | Don’t need this or N/A |
| --- | --- | --- | --- | --- | --- | --- |
| Did you have enough time to ask questions/voice concerns? |  |  |  |  |  |  |
| Did your clinician(s) listen carefully to concerns related to your health that may be related to cancer after treatment? |  |  |  |  |  |  |
| Did your clinician(s) show respect for what you had to say about follow-up care? |  |  |  |  |  |  |
| Did your clinician help figure out reasons for any new health care problems and whether they are related to cancer? |  |  |  |  |  |  |
| Did your clinician(s) explain things about cancer follow up care in a way easy to understand? |  |  |  |  |  |  |
| Did your clinician(s) explain reason for medical tests related to follow up care? |  |  |  |  |  |  |
| Did all clinicians involved in your care know about any medication(s) you are taking? |  |  |  |  |  |  |
| Did all of your clinicians have your up-to-date medical files about your cancer care? |  |  |  |  |  |  |
| Were you aware or did you notice that you have a team of clinicians who all work together to address your follow-up health care? |  |  |  |  |  |  |
| Did you receive instructions on when and how to transition care from oncologist back to primary care provider? |  |  |  |  |  |  |

**3. Following your visit today:**

|  | Yes | No | Don’t know | Don’t need this – N/A |
| --- | --- | --- | --- | --- |
| Do you have a point of contact to answer questions/ concerns about your follow-up care? |  |  |  |  |
| Do you have a regular clinician/ place to get your healthcare needs met including follow-up care after your cancer treatment? |  |  |  |  |
| Can all of your clinicians access your medical records online or through an Electronic Health Record? |  |  |  |  |

**4. On an ongoing basis, do you:**

|  | Never | Rarely | Sometimes | Always | Don’t know | Don’t need this |
| --- | --- | --- | --- | --- | --- | --- |
| Have regular access to exercise and physical activity services? |  |  |  |  |  |  |
| Have regular access to nutrition and dietary services? |  |  |  |  |  |  |
| Have regular access to risk reduction programs (e.g. weight loss, smoking cessation)? |  |  |  |  |  |  |
| Have clinicians who can provide referrals for mental health effects related to cancer treatment? |  |  |  |  |  |  |
| Have support to manage roles and relationships with partner, family, and others? |  |  |  |  |  |  |
| Feel included in decision-making about your cancer-related follow-up care? |  |  |  |  |  |  |
| Feel informed about what to do every day to take care of your health and healthcare needs? |  |  |  |  |  |  |
| Feel you are in control of your health and were able to manage health care needs? |  |  |  |  |  |  |
| Have the option to remain under the care of your cancer doctor until when ready to move care back to your primary care doctor? |  |  |  |  |  |  |
| Feel like your cancer doctor stays informed of your health after you transfer care to the primary care doctor? |  |  |  |  |  |  |

**5. On an ongoing basis, do you feel like your clinicians:**

|  | Never | Rarely | Sometimes | Always | Don’t know | Don’t need this |
| --- | --- | --- | --- | --- | --- | --- |
| Share information with each other about follow up care? |  |  |  |  |  |  |
| Give you the names of doctors to make an appointment with if you need more follow up care? |  |  |  |  |  |  |
| Help you with follow through on recommendations for follow-up? |  |  |  |  |  |  |
| Decide with you when and how to transition from oncologist to primary care provider? |  |  |  |  |  |  |

**You are almost done with the survey. In order for us to better serve all people who have finished cancer treatment, please tell us a little bit about you.**

**6. What is your age? ___**

**7. What is your highest level of education?**

- No school
- Primary school
- Some high school
- High school graduate or GED
- Some college or technical school
- College graduate
- Post-graduate degree (Masters)
- Doctorate
- Prefer not to answer

**8. What is your current partnership status?**

- Single, never married
- Married or in a domestic partnership
- Separated
- Divorced
- Widowed
- Prefer not to answer

**9. Which categories best describe your race, ethnicity or origin?** (Check all that apply to you.)

- American Indian or Alaska Native
- Asian
- Black or African American
- Hispanic, Latino, or Spanish origin
- Middle Eastern or North African
- Native Hawaiian or Other Pacific Islander
- White
- Some other race, ethnicity, or origin, please specify: ___________________
- Prefer not to answer

**10. What is your current gender identity?** (Don’t worry if you do not understand all of these terms. Check all that apply to you.)

- Female
- Male
- Cisgender
- Transgender
- Genderqueer/Non-binary/Two-spirit
- Other (please specify: ________)
- Prefer not to answer

**11. Do you think of yourself as:** (Check all that apply to you.)

- Straight or heterosexual
- Bisexual
- Gay
- Lesbian
- Queer
- Other (please specify:________)
- Prefer not to answer

**12. Which cancers have you been diagnosed before?**

- Bladder Cancer
- Breast Cancer
- Gastrointestinal Cancers
- Gynecological Cancers
- Head and Neck Cancers
- Kidney Cancer
- Leukemia, Lymphoma, and/or Myeloma
- Lung Cancer
- Melanoma
- Pancreatic Cancer
- Prostate Cancer
- Skin Cancer
- Thyroid Cancer
- Other Cancer (Please Specify): ____________________________
- Don’t know

**13. What stage of cancer were you most recently diagnosed with?**

- Stage 0
- Stage I
- Stage II
- Stage III
- Stage IV
- Don’t know

**Thank you for taking time to complete this survey. We appreciate your, time and feedback.**

# **How to Use Results**

**Step 1: Individual Surveys Total Sum**

1. Aggregate all responses by indicating the total number of each response option for each question asked.
2. Highlight items where there are a high number of “No, Never/Never/No” or “Don’t Know,” responses.
3. Consider potential quality improvements for items with a high number of aggregate “No, Never/Never/No” or “Don’t know” responses.
